# Designing measures of complex collaborations with participatory, evidence-centered design

**DOI:** 10.3389/frma.2024.1210547

**Published:** 2024-08-12

**Authors:** Caitlin C. Farrell, William R. Penuel, Paula Arce-Trigatti, James Soland, Corinne Singleton, Alison Fox Resnick, Kristina Stamatis, Robbin Riedy, Erin Henrick, Stacey Sexton, Sarah Wellberg, Danny Schmidt

**Affiliations:** ^1^School of Education, University of Colorado Boulder, Boulder, CO, United States; ^2^Institute of Cognitive Science, University of Colorado Boulder, Boulder, CO, United States; ^3^National Network of Education Research Practice Partnerships, Houston, TX, United States; ^4^School of Education and Human Development, University of Virginia, Charlottesville, VA, United States; ^5^College of Education, Health, and Human Sciences, University of Nebraska Omaha, Omaha, NE, United States; ^6^Partner to Improve, Nashville, TN, United States; ^7^Sagefox Consulting, Amherst, MA, United States

**Keywords:** evidence-centered design, research-practice partnership, measurement design, evaluation, instrument design, collaboration

## Abstract

An increasingly popular form of collaboration involves forming partnerships among researchers, educators, and community members to improve or transform education systems through research inquiry. However, not all partnerships are successful. The field needs valid, reliable, and useful measures to help with assessing progress toward partnership goals. In this community case study, we present a participatory, mixed-methods approach for creating measures to assess the progress of education research-practice partnerships (RPPs). The case illustrates a novel approach to measurement design, driven by perspectives and feedback of over 300 members of 80 partnerships. As a result, the measures align with the values and practices of the very collaborations the measures were intended to assess.

## 1 Introduction

There is an increased focus in research, policy, practice, and community circles on the potential of collaborative research partnerships to tackle longstanding challenges in education (Collaborative Education Research Collective, 2023). However, determining the effectiveness of these collaborations remain difficult because the field lacks suitable measurement tools. Such measures must be sensitive to the relational and interactive nature of collaborative work; be applicable in multiple, varied contexts; and reflect the dynamic ways in which local conditions shape what is possible (Thomson et al., 2009; Joss and Keleher, 2011; Tigges et al., 2019; Coombe et al., 2020).

We present a community case study that illustrates a process to design measurement tools for one kind of complex collaboration: research-practice partnerships (RPPs). RPPs are an emergent field of long-term collaborations aimed at improving or transforming educational outcomes through research inquiry (Farrell et al., 2021). As a community case study, our goal is to describe and reflect on practices and processes aimed at supporting field-driven needs (Smith et al., 2016). This special issue on “Practicing Collaboration” offers a platform to share a novel approach to participatory measurement design, one that reflects the values and practices of the very collaborations the measures were intended to assess.

This project sits at the crossroads of multiple traditions and fields. It joins a growing literature base in evaluation that looks at relationships as an asset for effectiveness (Brinkerhoff, 2002; Bright et al., 2017; Pisacane and Tagliacozzo, 2023). It contributes to literature that identifies strategies for assessing the benefits of cross-sector partnerships (Masse et al., 2008; Koontz and Thomas, 2012; Van Tulder et al., 2016). It extends insights on the process and outcomes of collaborative research efforts, as well (e.g., Israel et al., 1998; Schulz et al., 2003; Lucero et al., 2016; Goodman et al., 2019; Luger et al., 2020; Wallerstein et al., 2020).

We first describe the context of education research-practice partnerships (RPPs) and the growing emphasis on assessing their effectiveness. We introduce our approach to measurement development, characterized by participatory, evidence-centered design (ECD). Evidence-centered design is a principled approach for designing measures and guidance for their use. Across its phases, ECD is driven by a clear understanding of what to measure (i.e., constructs); when and where measurement will occur; which items to employ for revealing constructs; and how to support valid interpretations of the resulting data. We describe a process of intentional integration of the perspectives of over 300 members from 80 RPPs into the ECD process and ways for leveraging multiple data sources and methods to capture the intricacies of RPP work. We conclude by outlining possibilities and challenges with such an approach.

## 2 Context

Research-practice partnerships (RPPs) represent a unique approach to collaboration where participants work together to address persistent challenges and systemic inequities in schools and communities through research. RPPs stand in contrast to traditional research endeavors, which tend to support a one-directional flow of knowledge *from* research *to* practice (Farrell et al., 2021). Unlike typical research projects where the focus and conduct of inquiry is driven by a select few, RPPs draw on the collective expertise of individuals with different perspectives, collaboratively shaping and generating knowledge based on jointly established research agendas. The field of RPPs share many of the same values as community-engaged scholarship, participatory research, and other forms of collaborative inquiry with families, educators, and community members (Diamond, 2021).

The field has started to articulate the overarching goals that RPPs hold. The RPP Effectiveness Framework developed by Henrick et al. (2017) serves as a valuable starting point, reflecting the perspectives of a wide array of RPP leaders of the time. This framework includes five dimensions: (1) Building trust and cultivating partnership relationships; (2) Conducting rigorous research to inform action; (3) Supporting the partner practice organization in achieving its goals; (4) Producing knowledge that can inform educational improvement efforts more broadly; and (5) Building the capacity of participating researchers, practitioners, practice organizations, and research organizations to engage in partnership work. Almost immediately, it became a framework that evaluators, researchers, and RPPs themselves began to use to think about local RPP dynamics (e.g., McGill et al., 2021; Scholz et al., 2021; Weddle et al., 2021).

As external funding for RPPs has grown, questions of their effectiveness have emerged as well. Increasingly, funding agencies and RPP leaders have asked evaluators to judge the merit or worth of specific projects undertaken by research-practice partnerships (Schneider, 2018). Partnerships themselves are keen to determine how their partnership is doing, to improve from within (e.g., Wentworth, 2018). Measurement tools that are valid and reliable can help expand the ways in which RPPs learn about the effectiveness of their partnerships.

However, designing measurement tools for research-practice partnerships (RPPs) presents unique challenges. One issue lies in gaining an understanding of what constitutes effective collaboration in diverse contexts. A first step involved the creation of the RPP Effectiveness Framework, which emerged from interviews with members of various RPP types that were prevalent at the time (Henrick et al., 2017). More recently, the field has witnessed the emergence of novel and hybrid RPP approaches, encompassing community-based partnerships, partnerships involving state agencies, and hybrids that defy easy categorization within the original typology of RPPs (Farrell et al., 2021). Any definition of “effectiveness” must remain sensitive to the diversity of RPP activities, the communities they engage, and the objectives they pursue.

Further, a design process for measurement tools driven solely by measurement experts is insufficient within the RPP context. RPPs share a commitment to involving partners in crucial aspects of research, and in some projects, this commitment extends to the selection or creation of measurement tools. It would be inappropriate—and likely invalid—for an external entity to dictate the measures of effectiveness without involving key stakeholders in the development process. What is needed are participatory approaches that actively engage partnership members, representing various roles within RPPs and spanning diverse partnerships, in the tool development process (see, for example, Mark and Shotland, 1985; Ayers, 1987; Randall et al., 2022).

The complexity of collaborative work also demands an approach that deliberately plans for the integration of information from multiple sources. Collaborative or partnership work is inherently characterized by its relational, dynamic, and interactive nature (Gadja, 2004). Achieving determinations at the “RPP level” requires considering the viewpoints of multiple partners, whether across various research and practice-side members or those centrally or peripherally involved in partnership activities (Farrell et al., 2021). These dynamics may also require openness to various data collection and analysis methods, such as interviews, surveys, observations, artifact analysis, or other approaches, depending on the most appropriate or feasible sources of information (Lucero et al., 2016).

The validity of measurement is not solely determined by producing instruments with high reliability, nor is it sufficient for domain experts to agree that an instrument captures essential aspects of the phenomenon. The validity of measures is intrinsically linked to how the measures will be employed, including how the evidence is interpreted to draw conclusions from data and make decisions about future actions (Shepard, 1997). It also involves considering how measures can be appropriately used or misused and how the design of tools and processes can aid in shaping interpretation and sense making within local contexts (Ahn et al., 2019; Parker et al., 2020; Ing et al., 2021; Takahashi et al., 2022). In this context, we aimed to develop tools for low-stakes, formative use to assist RPPs in assessing their progress relative to broader RPP objectives.

## 3 Key elements of evidence-centered design

We draw on *Evidence-Centered Design* (ECD) as a promising approach to principled measurement design that can be adapted to the context of education RPPs (Mislevy et al., 1999, 2003; Mislevy and Haertel, 2006). In ECD, the primary objective is to develop a credible and compelling argument about the appropriate interpretation and use of evidence obtained from the assessments. ECD has been used in a range of settings, including the design and evaluation of tests of student learning (e.g., Mislevy et al., 2017; Arieli-Attali et al., 2019) and survey measures (e.g., Maul et al., 2016).

**Figure 1:**
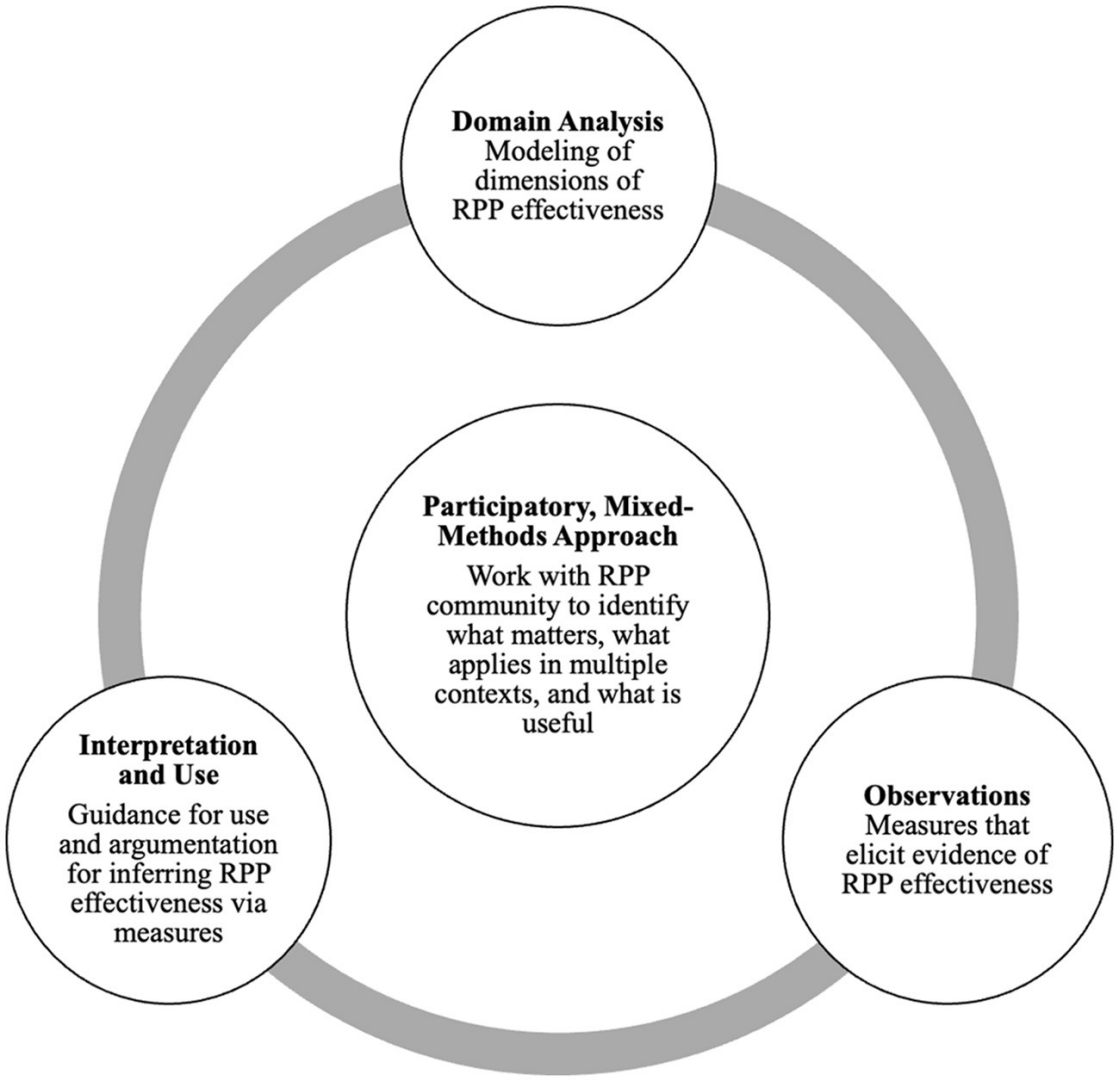
Participatory, evidence-centered design model.

ECD follows a “validity by design” approach, guided by three core questions: What constructs need to be measured? What behaviors or performances are indicative of, or demonstrate, those constructs? What tasks or situations elicit those behaviors? (Messick, 1994, p. 17). Typically, ECD involves three sets of activities, as seen in Figure 1. First, a team begins by consulting existing research, past measures and tasks, and input from relevant groups to describe constructs of interest (Domain Analysis). Next, the team selects or creates tasks to gather observable evidence of desired attributes for those constructs (e.g., valued activities, dispositions, or skills). Finally, the team analyzes how well the test tasks produce measurable, valid, and consistent evidence for the construct of interest (Observations) and determines what kind of guidance supports sense-making and interpretation of results for different purposes (Interpretation and Use). With the documentation produced during the ECD process, the team can then adjust these different components transparently and systematically, including the construct descriptions, the measurement tools, the collected observable evidence, and the interpretation of codes or scores. ECD unfolds iteratively, with measurement instruments being refined as new insights emerge from the performance of items or tasks (e.g., Mislevy et al., 1999).

ECD represents a different approach than traditional measurement development. Traditional methods often begin with a search for existing items or scales related to a broad construct and assembling possible items into a test or survey to pilot. Data from pilots and field tests are reviewed for evidence that the responses to items cohere in ways that are sensible to the assessment developer. Typically, questions of validity and reliability are determined based on psychometric analysis related back to the instrument tools themselves alone. ECD represents a significant advancement over these methods, both because it does not rely solely on existing evidence for construct definition and because it minimizes the trial-and-error approach that often consumes a considerable amount of time and resources. ECD is also distinct in the up-front investment of time in defining constructs and in the back-end approach to evaluating measurement tools within the context of their use.

Our use of ECD has several unique features. First, we drew on a mixed-method framework, employing ECD to design a set of survey measures and interview protocols with the aim of generating data that could support inferences regarding an RPP's alignment with the shared goals within the field. Second, our adaptation of ECD was intentionally participatory, emphasizing the involvement of RPP members throughout every stage of the process, including the development, testing, and refinement of constructs, instruments, and usage guidelines. Not all ECD involves the direct participation of interest holders in design, as we did (although due to confidentiality constraints, we are unable to disclose the names of individuals or partnerships involved). Finally, we took great care to consider various use scenarios, and we crafted, tested, and improved multiple tools and procedures to facilitate local interpretation and reflection. Together, the resulting measures and tools are designed to produce data that can serve as evidence for assessing where an RPP stands in relation to its goals, while remaining attuned to the fluid and diverse nature of collaborative efforts.

### 3.1 Domain analysis

The initial phase of ECD involves conducting a domain analysis to determine what needs to be assessed. This phase entailed collecting information related to the primary outcomes of RPPs, as well as the methods, timing, and locations where these outcomes could potentially be observed. We drew on participant perspectives in interviews and a convening where we assembled leaders from various RPPs. This phase also included a review of research related to RPP outcomes and conditions for their success and an analysis of existing measures of collaborative research (Farrell et al., 2021).

#### 3.1.1 Interview study

In 2019, members of the project team conducted interviews with a total of 29 leaders representing 16 different educational RPPs located across the United States. The primary aim of these interviews was to update the descriptions of outcomes outlined in the RPP Effectiveness Framework developed by Author and colleagues in 2017. This update encompassed individuals and groups who had not participated in the initial creation of the framework based on field research in 2017.

To identify the specific stakeholders for these interviews, we employed purposeful sampling techniques as recommended by Palinkas et al. (2015). We accessed lists of partnerships from six online sources, including websites of funders and networks of RPPs. Our objective was to ensure maximum diversity in terms of partnering approaches and roles within partnerships.

As depicted in Table 1, this group included representatives from the original RPP typology, consisting of seven design partnerships, six research alliances, and four networked improvement communities. Additionally, we involved leaders from community-based partnerships, partnerships involving state agencies, partnerships linked to Regional Educational Laboratories, and hybrid partnerships that did not neatly fit into any of the original typology categories. Among the interviewees, 18 individuals identified themselves as research-side partners, while 11 identified themselves as practice- or community-side partners.

**Table 1:** Interviews by role and RPP experience for domain analysis.

		***N***	**%**
Role	Research-side	18	62
	Practice-/community-side	11	38
RPP experience^*^	Design-based partnerships	7	24
	Hybrid	7	24
	Research alliance	6	20
	Community-based partnerships	5	17
	Networked improvement community	4	14

^*^Role and RPP experience “type” are participants' self-reports.

During these interviews, we invited leaders from various RPPs to engage in reflective discussions regarding the existing descriptors of the five outcomes outlined in the RPP Effectiveness Framework. In each interview, RPP members were encouraged to offer their interpretations and critiques of these dimensions, either in the context of their own RPP or by considering RPPs more broadly. For each of the outcomes, we requested that these leaders provide descriptions of specific scenarios in which the outcome could be observed and share examples from their own partnerships that illustrated such situations.

We engaged in thematic analysis of these interviews to understand possible areas for change or exploration in the existing framework (Miles and Huberman, 1994). Overall, interviewees suggested that the same five dimensions reflected the desired goals for partners, and that the dimensions should remain relatively general to accommodate for variation in RPP approaches and goals. One key idea was the need to more explicitly call out and understand how RPPs attended to equity with respect to each outcome. As one research-side partner expressed:

I've started to come to believe honestly too, that with a lot of the problems that our partners in schools are facing, is the equity part of the conversation. I really think that's part of a healthy partnership as well. Are they [RPP members] able to talk about that, and do they have tools to interrogate that?

These interviews coincided with a rising national urgency to confront issues of inequity, systemic racism, violence against Black, Asian, and LGBTQIA+ communities, and racialized xenophobia within the United States. Building on this theme and relevant literature (e.g., Diamond, 2021), we developed plans for designing complementary equity strands for each dimension. A second theme from the interviews was to shift from language of “researcher” and “practitioner” as set roles toward a broader range of possible participants, with the view that these roles can be fluid.

#### 3.1.2 Convening

In 2020, we brought together RPP representatives and measurement researchers to aid in the development of a particular type of item blueprint used in ECD known as a “design pattern.” In ECD, a design pattern serves as a template for crafting test or survey items, observation protocols, or interviews that can be used by a variety of designers to guide development (Mislevy et al., 2003). Though design patterns can be time consuming to generate, they can improve the likelihood that measures developed will elicit what is intended. Moreover, when collaboratively generated by those with in-depth knowledge and expertise of RPP work, they constitute a form of validity evidence regarding how constructs to be measured are represented.

We collaboratively design patterns for each of the five dimensions of effectiveness, involving individuals deeply engaged in the RPP field. The 27 participants represented diverse backgrounds, with 12 actively involved in different types of partnerships, six primarily identifying as researchers or evaluators of research-practice partnerships, six possessing expertise in survey design, research utilization, and psychometrics, and three representing funders. Each participant was assigned to a role-diverse group, focusing on one of the five dimensions outlined in the RPP Effectiveness Framework, to collectively create design patterns for each outcome.

**Figure 2:**
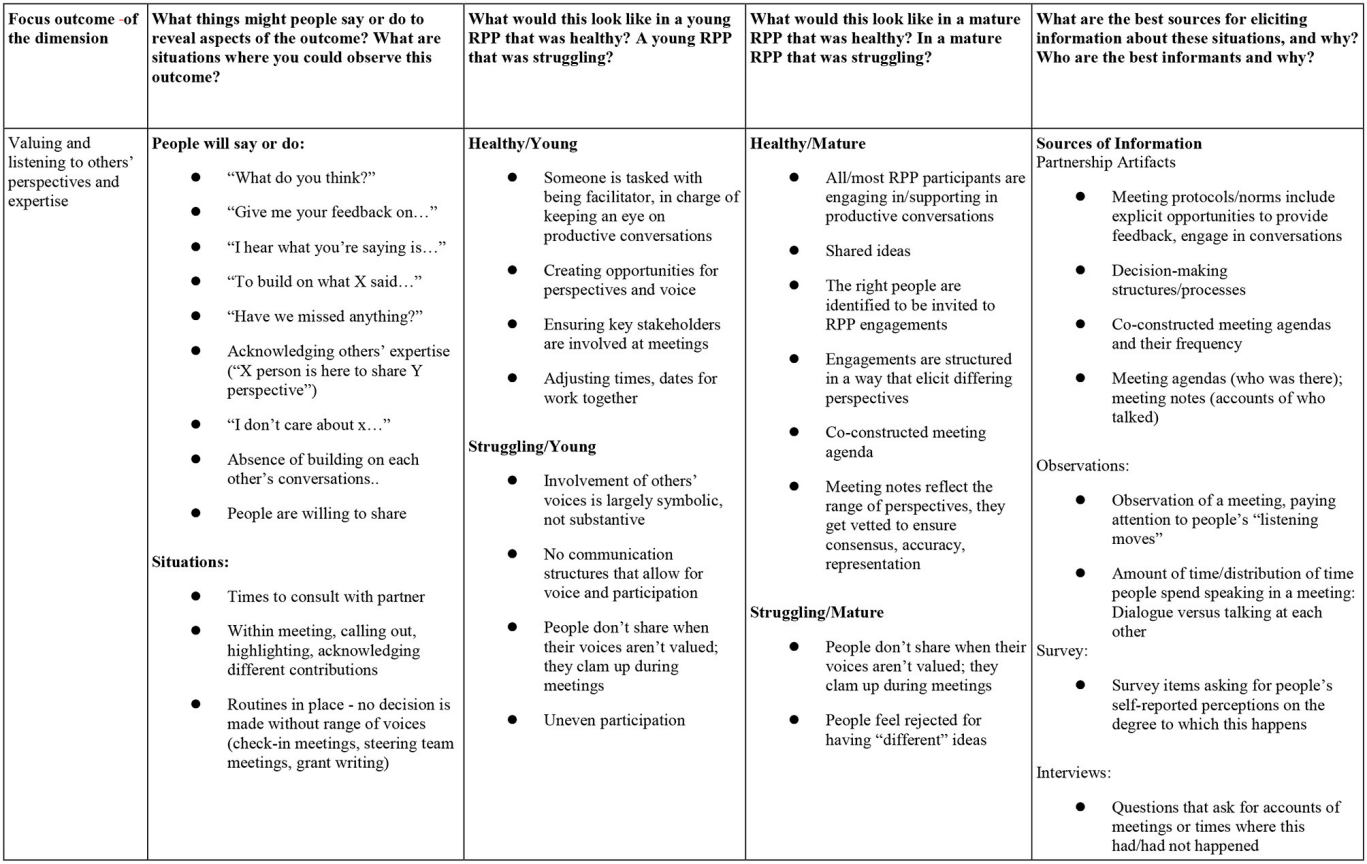
Ideas developed during convening related to Dimension 1, building trust and cultivating relationships.

Drawing on the insights from earlier interviews, the groups reviewed and revised paragraph descriptions of each dimension. They also considered how an RPP could address equity concerns within the context of each outcome. Subsequently, the groups broke down each dimension into essential indicators. For each indicator, the group identified what things people might say or do to reveal that aspect of the outcome, situations where the aspect could be observed, what the best sources of data for eliciting information about the situations were, and why, as well as who the best informants were, and why. Within and across groups, individuals pushed one another to think about where and how the dimensions of effectiveness would manifest in different settings. For example, for each dimension, groups identified what an RPP in early stages of progress on a dimension might look like in contrast to an RPP more mature in that area. Groups also began developing possible survey items and interview questions to effectively capture relevant information. Figure 2 is an illustrative example of the work product that emerged from the co-design activities during this convening.

Instead of starting with a specific measurement tool to be designed, the convening enabled us to develop ideas about what the dimensions might look like from multiple perspectives within an RPP and across different approaches to RPP work. We gained a deeper comprehension of the dimensions themselves and a field-driven collection of insights into the range of scenarios and the wide variation in potential data sources (e.g., surveys, interviews, and observations) that could be employed to provide pertinent information. We also drew on these insights to design draft developmental trajectories for possible qualitative differences for emerging, maturing, and sustaining partnerships in each area.

### 3.2 Tool design and testing

Acknowledging that it was not feasible to create validated measurement tools for every data collection type covering all dimensions outlined in the design patterns, our focus shifted toward developing and improving measures that could be widely applicable across a variety of RPPs and relatively straightforward for RPP members and evaluators to use. One key area of subsequent effort was the development and testing of a pilot survey, which encompassed claims across the five dimensions and drew upon the design patterns established in the earlier phase.

#### 3.2.1 Cognitive interviews

A challenge to developing valid measures is that individuals may not always understand questions in the same way that designers intend. In a relatively new field such as research-practice partnerships, even widely used terms such as “partner” may mean different things to different people. Cognitive interview studies are a strategy for refining the wording of items so that people from varied backgrounds are able to interpret questions in the ways they are intended (Desimone and Le Floch, 2004). We purposefully selected individuals for partnership and role diversity to represent the broadest range of perspectives.

In the first round, participants reviewed revised dimensions, claims, and hypothetical developmental progressions for each dimension in addition to sample items. In the second round, participants simultaneously completed sets of survey items and “think aloud” interviews about the basis for their answers. In all, 23 of the 28 people we invited (or 82%) participated in cognitive interviews (see Table 2).

**Table 2:** Roles and RPP types for cognitive interviews.

		***N***	**%**
Role	Research-side	13	57
	Practice-/community-side	7	31
	Broker or evaluator	3	13
RPP type	Experiences in multiple RPP approaches	7	30
	Networked improvement community	5	22
	Research alliance	4	17
	Community-based partnerships	4	17
	Design-based partnerships	3	13

Participants in this stage represented the primary end-users of the measures and tools, making this phase crucial for refining the instruments. One objective was to assess whether potential respondents interpreted the questions in a manner consistent with the developers' understanding of the claim and how that idea had been communicated within a specific item.

For instance, concerning Dimension 1, which pertained to building trust and nurturing relationships, an initial item asked respondents to indicate their agreement (using a Likert scale) with the statement: “Partners are able to resolve conflicts effectively when they emerge.” During a cognitive interview, a partner from an RPP pointed out that this wording “…assumes that partners are effectively bringing conflict to the forefront, but if conflict doesn't arise, it could signal a reluctance to do so.” Consequently, we modified the item to better capture this nuance: “Partners feel comfortable discussing an issue when a conflict arises.” While this change may seem minor in terms of wording, it had a substantial impact on the item's meaning, rooted in the practical experiences of partnership work, where the absence of conflict resolution efforts can signify an underlying lack of trust or mutual confidence.

The feedback from these interviews led to more holistic adjustments. Specifically, items related to equity needed rephrasing to ensure relevance for RPP members across various approaches and types of partnerships. During the revisions, we provided clarity within items by explicitly defining terms such as “status,” “power,” “equity,” “equitable participation,” and “shared values.” Responding to the recommendations of RPP members, we also refined the language to be as specific as possible to minimize confusion for survey participants. Additionally, feedback from RPP members revealed that our use of the term “partner” was not consistently clear. In the revised survey version, we provided distinct definitions for “partner,” indicating that it referred to someone representing either an organization or a community involved in the RPP, distinguishing it from “stakeholder,” which referred to individuals or groups with a vested interest in the focal issue but who might not be actively engaged in the partnership.

#### 3.2.2 Pilot testing

We conducted a pilot test of the survey, with participants recruited from the annual meeting of the National Network of Education Research-Practice Partnerships, totaling 28 participants. During this testing phase, a new issue emerged: how to solicit responses from partners when there were multiple projects or lines of work within an RPP? On one hand, asking partners to respond in reference to the RPP could provide a solid basis for making evaluative judgments at the RPP level. However, pilot test respondents informed us that it was overly challenging to respond in a generic manner when multiple lines of work existed, each potentially warranting different evaluations.

Based on this feedback, for RPPs involving multiple projects and lines of work, respondents would be encouraged to consider a project that (1) embodied the core objectives of the partnership and (2) was of particular interest to the RPP members. This approach meant that the inferences drawn from the data would now pertain to the project rather than to the RPP as a whole.

#### 3.2.3 Field test

From the earlier efforts, we recognized the importance of deliberately involving a sample of RPPs that represented the diversity of approaches within the field. We constructed a national census encompassing active, externally-funded education RPPs, drawing from grant funding sources and empirical RPP scholarship from 2013 to 2020. This census resulted in a list of 303 RPPs in total.

For the purpose of creating a purposeful and stratified sample, we opted to consider both the age and “type” of RPPs. These types included Networked Improvement Community, Research Alliances, Community Based Participatory Research, Design-Based Implementation Research, or a hybrid of two or more of these types. RPPs exhibit a wide range of organizational structures, and the typology used to categorize them, outlined by Coburn et al. (2013), is one that many researchers and evaluators have frequently referenced (e.g., Ballard et al., 2020). RPPs themselves employ this typology to characterize their partnerships (e.g., Kali et al., 2018). Following communication with the leadership of each RPP and inviting their participation, we ultimately included a total of 65 RPPs in the sample (see Table 3).

**Table 3:** Sample of partnerships by type and age in field test survey.

	**Alliance**	Design^*^	**Networked improvement community**	**Community**	**Total**
Older, >3 years	9	6	8	4	27
Younger, 1–3 years	8	11	9	10	38
Total	17	17	17	14	65

^*^Includes RPPs that self-identified as hybrid design/community.

We had learned from our earlier phases that selecting informants who can give a good “read” on the partnership and its effects was a challenging but critical issue. To assemble a sample of informants with a deep understanding of the day-to-day operations of the partnership within a specific project of the RPP, we requested principal investigators or directors from each RPP to nominate a minimum of two individuals from each partner organization for participation. In order to ensure a diversity of perspectives, we aimed for participation that included a minimum of two individuals from the research-side and two from the practice- or community-side within each RPP. The full participant pool consisted of 285 members representing 65 RPPs who completed the survey (details available in Table 4). For approximately half of these RPPs (*n* = 32), 132 individual RPP members also participated in interviews.

**Table 4:** Field test survey participant demographic information.

	**All**		**Research-side**		**Practice-/community-side**	
	***N***	**%**	***N***	**%**	***N***	**%**
**Race/ethnicity**						
Asian	20	7.0	13	8.8	7	5.1
Black	13	4.6	4	2.7	9	6.6
Latinx	24	8.4	9	6.1	15	10.9
Multiple	2	0.7	1	0.7	1	0.7
White	207	72.6	115	77.7	92	67.2
Left Blank	19	6.7	6	4.1	13	9.5
**Gender**						
Female	205	71.9	109	73.6	96	70.1
Male	80	28.1	39	26.4	41	29.9
**Education**						
Some College	1	0.4	0	0.0	1	0.7
BA	26	9.1	4	2.7	22	16.1
MA	99	34.7	28	18.9	71	51.8
Doctorate	140	49.1	114	77.0	26	19.0
JD	1	0.4	0	0.0	1	0.7
MD	1	0.4	0	0.0	1	0.7
Other	12	4.2	2	1.4	10	7.3
Left Blank	5	1.8	0	0.0	5	3.6

Engaging with RPP members during the field test phase was essential for gaining insights into the feasibility and effectiveness of the designed survey items (for a more comprehensive discussion of the survey field test). In a broad sense (see Table 5), our analysis revealed that the scales were generally reliable. Additionally, our Evidence-Centered Design (ECD) and theory-based conceptualization of the constructs being measured are largely aligned with the item response data. However, a noteworthy finding was that the functioning of items exhibited significant differences between practice- and community-side partners on one hand and research-side partners on the other. This observation suggests that the perspectives of these two groups on partnership may fundamentally differ or that they interpreted the items in distinct ways.

**Table 5:** Factor analytic and reliability results for each dimension.

	**Dimension**				
	**1**	**2**	**3**	**4**	**5**
Hypothesized factors	3	2	4	1	3
**Confirmatory factor analysis (CFA) results**					
**Hypothesized factors**					
RMSEA	0.097	0.099	0.083	0.197	0.036
CFI	0.985	0.964	0.978	0.925	0.986
**Reduced factors**					
Factors	2				
RMSEA	0.074				
CFI	0.992				
**Exploratory factor analysis (EFA) results**					
**Eigenvalues**					
5 factors	0.279	0.167	0.771	−0.200	0.385
4 factors	0.529	0.384	1.017	−0.123	0.565
3 factors	0.724	0.758	1.635	0.053	0.768
2 factors	1.207	1.211	2.040	0.478	1.267
1 factor	6.965	4.060	6.562	1.937	4.636
**Reliability estimates**					
**Alpha/omega**					
Claim A	0.83/0.90	0.73/0.85	0.66/0.73	0.74/0.81	0.77/0.88
Claim B	0.89/0.94	0.80/0.86	0.82/0.91		0.58/0.75
Claim C			0.86/0.94		0.79/0.85
Claim D			0.88/0.92		

### 3.3 Interpretation and use

Any development of measures requires careful attention to the different purposes for which various users might apply them (Kane, 2006; American Educational Research Association, 2014). We continued to engage the voices of RPP members and evaluators to help us refine measures and to develop and test strategies to support sense-making and interpretation of data.

#### 3.3.1 Mixed-methods approach to validity

In our approach for analysis, we prioritized the end-user perspective. We integrated psychometric data with emerging themes derived from interviews with members of the RPPs and evaluators when making final decisions regarding refinements to our construct claims and measurement tools. To ensure a comprehensive analysis, we adopted a mixed-methods approach, assessing the convergence and divergence of insights from these diverse sources (Campbell et al., 2020). For the purpose of revising and validating our measures, we conducted an analysis of the interview data, focusing on respondents' interpretations of the claims and their relevance to their partnership experiences. We then combined these insights from the interview validity analysis with those obtained from the psychometric analysis of survey data. We balanced findings from both types of validity analysis, revising our scales to incorporate insights from both sources. Subsequently, we conducted retests to ensure that the revised scales maintained acceptable psychometric characteristics.

In terms of the survey scales, we found that RPP effectiveness scales had strong internal consistency, and the data provided support for our theories about what each scale was measuring. However, given limited within-RPP sample sizes and measurement inconsistencies by respondent type, we cannot yet recommend use of these scales to make judgments about the efficacy of a given RPP, especially for consequential purposes. Consistent with good evaluation practice, use of the survey should include other measures and sources of evidence to draw inferences about RPP effectiveness.

#### 3.3.2 Insights from RPP evaluators

Many RPPs include individuals who serve in an evaluation capacity, assisting specific RPPs in their improvement efforts. We conducted interviews with eight RPP evaluators to gain insights into their approaches, strategies, and best practices in the realm of RPP evaluation. Our team also organized and led two workshops involving over 30 RPP team members, comprising practice-, community- and research-side partners, as well as RPP evaluators. These workshops provided valuable insights into methods of gathering, interpreting, and sharing RPP evaluation data.

Through these engagements focused on RPP evaluation, several key lessons emerged. One was the significance of RPPs establishing a regular schedule for their evaluation activities, be it monthly, quarterly, or annually. Furthermore, RPP teams and RPP evaluators emphasized the need to develop a range of adaptable tools and approaches tailored to the specific goals, contextual conditions, and challenges of each RPP. A final notable theme underscored the importance of creating structures that facilitate RPP learning through meaningful sense making and dialogue around data while elevating diverse voices. This outcome could be achieved by incorporating discussion questions that encourage conversation and the empowerment of participants to determine their next steps.

#### 3.3.3 Tool design and refinement

We developed a “package” for each of the five dimensions from the RPP Effectiveness Framework which included a description of the dimension and key claims; examples of variation for each claim; related survey scales (1–2 per dimension); use and interpretation guidance for the survey scales; and facilitated discussion activities to facilitate sense making, interpretation, and learning within each dimension (Henrick et al., 2023; National Network for Education Research-Practice Partnerships, 2024). All tools were designed around key principles that surfaced and were reinforced from the earlier phases. For example, meaningful evaluation of RPP effectiveness should involve the participation of members of the RPP team and draw on multiple sources of data, as each can provide different insights on the work. Further, efforts to support formative improvement efforts for RPPs will likely require ongoing reflection, communication, and learning with careful attention to the unfolding partnership dynamics, context, and work of the RPP. As with earlier phases, all activities and tools have been further refined through prototype testing with RPP evaluators, individual RPPs, and groups of RPPs.

## 4 Discussion and limitations

This community case study presents a participatory, mixed-methods approach to evidence-centered design for measures of complex collaboration. Our primary focus is on education RPPs, which serve as an example of complex collaborative efforts centered around the investigation of local issues. The process was anchored in developing a deep understanding of the construct-level claims, design and testing cycles to develop and validate measures that would elicit evidence for these claims, and development of structures and guidance to support their use by RPPs.

Central to this endeavor were the perspectives, needs, and insights of the RPP community. We actively engaged with hundreds of RPP members in various capacities throughout the project: as thought partners, co-designers, field test participants, tool testers, and as experts at their local sites where future adaptation and experimentation would take place. Also key to the work was an openness to bringing together and learning from multiple sources of information, including survey scores, interview themes, and the insights from individuals and teams actively engaged in RPP evaluation work.

This process was not without its challenges. Descriptions of dimensions and claims, tools, and guidance evolved over time and across different groups involved, sometimes leading to tensions. Rather than viewing these tensions as flaws, we embraced them as integral to the co-design process, recognizing that they emerged as various groups converged around different ideas, aiming to move forward in ways that honored earlier thinking while incorporating new perspectives.

Looking ahead, there is a clear need for further critical reflection concerning RPP effectiveness measures and tools, particularly in a way that encompasses diverse perspectives on what constitutes “success” and multiple ways of understanding it, particularly in ways that center the perspectives of historically marginalized groups (Denner et al., 2019; Tanksley and Estrada, 2022; Villavicencio et al., 2022). Also, our design patterns specified a number of things RPP members might say or do that could be elicited through a range of methods, including observations or artifact analysis, which we did not develop because of limited resources. Other design teams can use the design patterns to develop additional measures. Finally, future work should investigate if and how RPP teams engage with, adapt, and use these tools in diverse environments over time, and with what consequences.

Overall, the work of RPPs—including the evaluation and assessment of their effectiveness—is inherently complex, rooted in local conditions and shaped by specific goals, approaches, and relationships. Any attempt to develop assessments for gathering valid information about such efforts must acknowledge this complexity. This participatory, mixed-methods approach to developing evaluative measures provides one way to do so, with a strong emphasis on how conclusions are developed, interpreted, and used by those who will act on them (House, 1977). It represents an innovative approach to assessment design, one that mirrors the values and practices of the very collaborations the measures were designed to assess.

## Data availability statement

The original contributions presented in the study are included in the article/supplementary material, further inquiries can be directed to the corresponding author.

## Ethics statement

The studies involving humans were approved by University of Colorado Boulder Institutional Review Board. The studies were conducted in accordance with the local legislation and institutional requirements. The ethics committee/institutional review board waived the requirement of written informed consent for participation from the participants or the participants' legal guardians/next of kin because we had approval for gathering and documenting verbal consent.

## Author contributions

CF, WP, PA-T, and JS contributed to the conception and design of the project. CF and WP wrote the first draft of the manuscript. All authors were involved in one or more phases of the Evidence-Centered Design process, contributed to manuscript revision, and approved the submitted version.
